# Temporal muscle thickness predicts change in nutritional markers in individuals at risk of dementia: Insights from a 24-week longitudinal study

**DOI:** 10.1016/j.jarlif.2025.100023

**Published:** 2025-08-08

**Authors:** Salomón Salazar-Londoño, Valeria Pérez-Foucrier, Jonathan Patricio Baldera, Markus Aarsland, Luis Carlos Venegas-Sanabria, Miguel German Borda, NJ FINGER

**Affiliations:** a Semillero de Neurociencias y Envejecimiento, Aging Institute, Medical School, Pontificia Universidad Javeriana, Carrera 7#62-43, 110231, Bogotá, Colombia; b Centre for Age-Related Medicine (SESAM), Stavanger University Hospital, Gerd-Ragna Bloch Thorsens gate 8, 4011, Stavanger, Norway; c Instituto de Investigación en Salud, Facultad de Ciencias de la Salud de la Universidad Autónoma de Santo Domingo, *Av*. Alma Máter, 10105, Santo Domingo, República Dominicana; d Hospital Universitario Mayor – Méderi, Calle 24 #29-45, 111711, Bogotá, Colombia; e Rosarist Institute for the Study of Aging and Longevity, Universidad del Rosario, Carrera 24 #63C-69, 1112, Bogotá, Colombia; f Centro de Investigación en Ciencias de la Salud (CICSA); FCS, Universidad Anáhuac México Campus Norte, Avenida Universidad Anáhuac número 46, 52786, Huixquilucan Edo. De México, Mexico; g Department of Neurology, Clínica Universidad de Navarra, Avenida Pío XII 36, 31008, Pamplona, Spain

**Keywords:** Cognitive dysfunction, Heart disease risk factors, Temporal muscle, Nutritional status

## Abstract

**Objectives:**

To investigate whether temporal muscle thickness (TMT) can serve as a predictor of change in nutritional widely used biomarkers in patients at risk of dementia.

**Design:**

Longitudinal observational study.

**Setting:**

Secondary analysis from a 24-week study conducted across three centers in Norway between 2018 and 2020.

**Participants:**

Patients with mild cognitive impairment (MCI) or with two cardiometabolic disorders were included (*n* = 165).

**Measurements:**

Baseline and longitudinal statistical analysis were carried out to establish the association for outcomes (albumin, weight, C-Reactive Protein and Episodic Memory Quality) with TMT.

**Results:**

At baseline, there was a positive association between TMT and weight (Estimate=1.5157, *p* = 0.009). At follow-up, positive associations were observed between TMT and albumin levels (Estimate=0.3031, *P* = 0.048), as well as TMT and weight (Estimate=1.8954, *P* = 0.001).

**Conclusion:**

TMT is a possible accessible tool in clinical practice for monitoring health variables beyond cognitive decline in patients at risk of dementia.

## Highlight

• In patients in the spectrum of cognitive decline, it is important to consider outcomes beyond cognition, such as nutritional status.
• At follow-up, temporal muscle thickness was associated with albumin levels and weight.
• Temporal muscle thickness is a widely available biomarker, that could possibly be a tool for the assessment of patients beyond cognition.

## 1 Introduction

Dementia is becoming one of the most pressing public health issues worldwide, with its prevalence projected to rise substantially in the coming years [1]. Although there have been important advances in understanding its biological mechanisms, effective disease-modifying treatments remain limited. This situation highlights the urgent need to identify individuals at risk and intervene before significant and irreversible decline occurs. Aiming to diagnose the disease as early as possible, current efforts to promptly identify Alzheimer’s disease (AD) have largely relied on a biological framework [2]. However, this approach tends to overlook other key dimensions of prognosis that go beyond diagnosis and cognitive decline.

People living with dementia are at higher risk of experiencing a range of conditions, such as falls, sarcopenia, frailty, loneliness, malnutrition, and dysphagia, among many others. These conditions ultimately impact the individual's functional performance, independence and quality of life [3,4]. For example, malnourished older adults with dementia have faster functional decline and have higher mortality compared with those with dementia, but not malnutrition [5]. In this context, timely assessments that capture factors beyond cognitive decline, such as nutritional status, are essential for comprehensive care [6].

Nutrition and muscular mass have recently been recognized as important prognostic markers in older adults, including those with chronic obstructive pulmonary disease, cancer, and dementia, within others [5,7,8]. These factors share biological mechanisms including inflammation, metabolic dysregulation, and low physical activity, particularly relevant for those living with the mentioned conditions. Measuring muscle mass in older adults and preventing sarcopenia are emerging as key strategies, especially for individuals with a high burden of comorbidities.

Following this thought, temporal muscle thickness (TMT) has been shown to be a promising marker for muscle mass assessment, being practical and easily applicable in the clinical setting because it utilizes routinely requested head magnetic resonance imaging (MRI) [9]. TMT has been associated with important clinical outcomes, such as frailty, disability, and mortality in older adults, including those with cognitive decline [10]. Nonetheless, there is limited evidence on its potential value in individuals at risk of dementia or as an early signal of nutritional vulnerability.

This study aims to evaluate whether TMT is associated with deterioration in nutritional biomarkers among individuals at risk of dementia. Our hypothesis is mechanistically grounded and draws on well-established physiopathology that links nutrition, systemic inflammation, and muscle loss. We propose that lower TMT reflects early systemic vulnerability, which may parallel or even precede clinical decline.

## 2 Materials and methods

### 2.1 Design

This secondary analysis examines data from a 24-week randomized, double-blind, placebo-controlled phase II trial conducted across three centers in Norway between 2018 and 2020, known as the "AnthoCyanins in People at Risk for Dementia" (ACID) study [11]. For the present manuscript, the exposure to purified anthocyanins was not considered in the analysis as an exposure variable. The trial was approved by the Norwegian Regional Ethics Committee (2017/374) and registered on ClinicalTrials.gov (identifier NCT03419039). Written informed consent was obtained from all participants in accordance with Good Clinical Practice guidelines prior to their inclusion in the study.

### 2.2 Study population

Participants were recruited through referrals from outpatient clinics specializing in geriatrics, psychiatry, neurology, cardiology, and memory, as well as through an ongoing cohort study, community outreach, and social media campaigns. Follow-up phone calls were conducted 4 weeks after enrollment, and participants attended clinic visits at 12 weeks and 24 weeks (final visit). They were advised to maintain their usual diet and lifestyle throughout the study period.

**Fig. 1:**
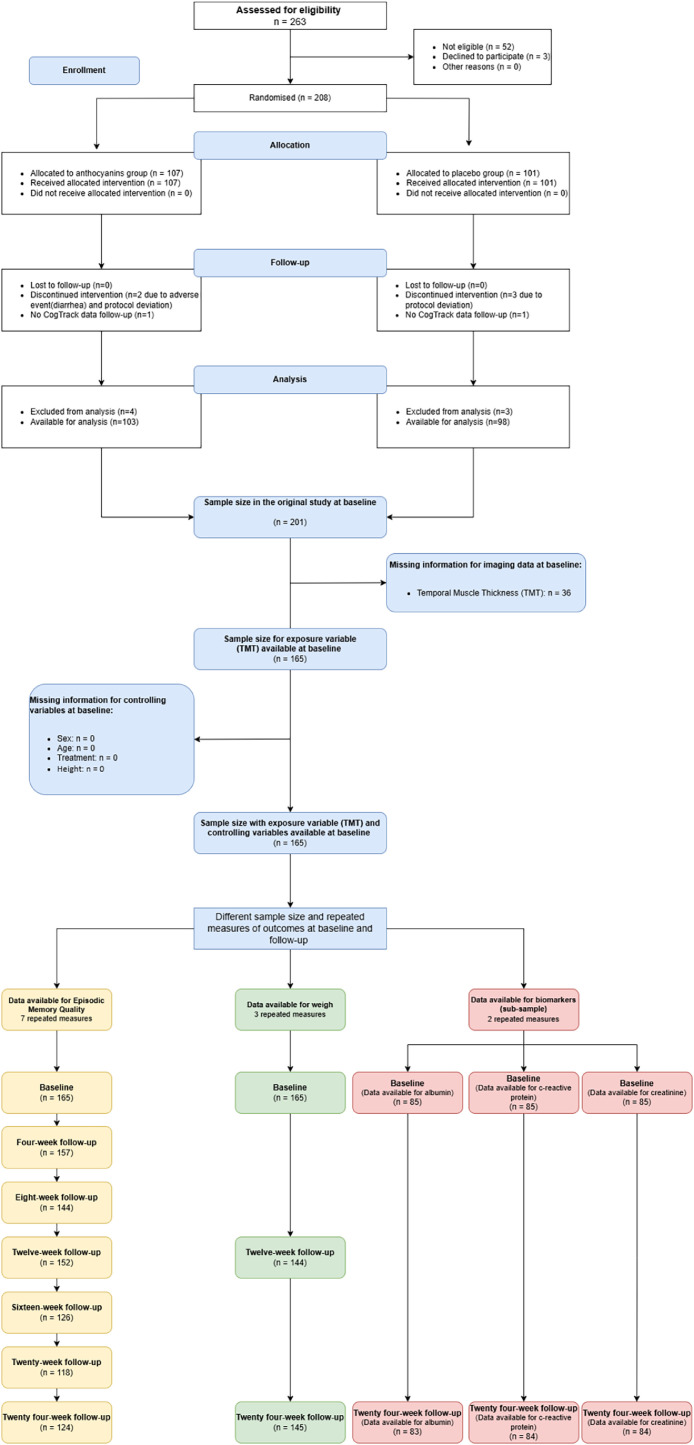
Sample selection and distribution.

The inclusion criteria for participants aged 60–80 years were as follows: a) they had mild cognitive impairment (MCI) according to Winblad et al. criteria [12], with or without cardiometabolic disorders, or b) they were cognitively healthy but had at least two cardiometabolic disorders, which are associated with a higher risk of cognitive decline and dementia. Exclusion criteria included a diagnosis of dementia, Parkinson’s disease, history of stroke within the past 5 years, other significant somatic diseases that could impair cognitive function as determined by the study physician, clinically significant depression, anticoagulant use, and any prior use of the investigational product within 12 months before inclusion. Additionally, individuals who had difficulty with computerized testing were excluded. A detailed list of inclusion and exclusion criteria and the complete study protocol are available elsewhere [13]. For each outcome a sample was established depending on the availability of data. Sample selection and distribution are presented in Fig. 1.

### 2.3 Imaging techniques

The structural 3D T1-weighted (T1w) imaging data were collected at 3 different sites in a period between May 2018 and October 2019. Stavanger University Hospital performed the imaging on Philips Ingenia 3.0T (Best, the Netherlands) utilizing a ds Head 32-channel coil. The sagittal 3DT1 FFE sequence were scanned with parameters: (TR = 7.9 miliseconds (ms), TE = 3.7 ms, FA = 8 degrees, 170 slices with a slice thickness = 1 mm, FOV = 240 mm, voxel size 1 × 1 × 1 mm3, acquisition time was 5 min and 40 s). On the other hand, Betanien Hospital in Bergen and Evidia Oslo City in Oslo, Norway, scanned the 3D T1w images on Siemens Avanto fit 1.5T (Erlangen, Germany) with a Head/Neck 20-channel coil. Sagittal MPRAGE T1-images were acquired with the following parameters: (TR = 1180 ms, TE = 4.18 ms and 3.92 ms, FA = 15 degrees, time of inversion = 905 ms, 160 slices with a slice thickness = 1 mm, FOV = 256 mm, voxel size 1 × 1 × 1 mm3, acquisition time was 6 min and 12 s).

Images underwent quality control, and those compromised by motion artifacts or poor quality were excluded. A standardized pre-processing procedure was applied, including movement correction and intensity normalization. The images that met quality standards were used to assess TMT.

### 2.4 TMT measurement

**Fig. 2:**
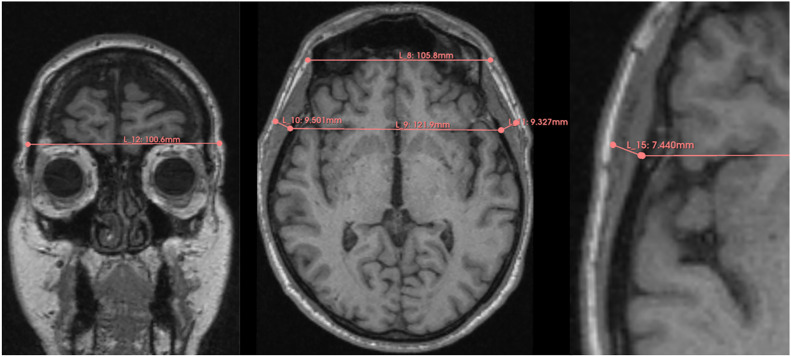
Temporal muscle thickness measurement.

Axial T1w images were analyzed to measure the thickness of the temporal muscle on both the right and left sides independently, perpendicular to the long axis of the temporalis muscle, with the Sylvian fissure and the orbit roof as reference points. An average TMT in mm was then calculated for each patient for subsequent statistical analysis (Fig. 2). A single trained researcher conducted the measurements, and to ensure consistency, 20 randomly selected images were re-measured to confirm high intra-rater reliability.

### 2.5 Variables

Blood samples were collected at weeks 2, 6, 12, and 24, and processed following specific protocols. Longitudinal comparisons were made for biomarkers, including C-reactive protein (mg/L) and albumin (g/L). Specifically for each variable, a subsample was assessed based on data availability, and Fig. 1 states the number of patients per outcome. In a comparison between the missing and the included subjects, no significant differences were found (Supplementary material 1).

Cognitive performance was assessed using the CogTrack® tool, an online cognitive assessment battery comprising 10 subtests. It is validated online and for multiple measurements in individuals from 50 up to 94 years old based on data from 14,531 participants [14]. Specifically for this study, the primary outcome measure was the quality of episodic memory composite score, which combines word and picture recognition outcome data from the CogTrack® at week 24. The range of values is 0–100 [11].

Other variables included were weight in kilograms (kg), height in centimeters (cm), age in years and years of education.

We chose the previously mentioned variables based on data availability, as this is a secondary analysis and data was not recollected with the purpose of the present study. Moreover, even if having composite measures as the mini-nutritional assessment or the global leader in malnutrition (GLIM) approach would enhance the quality of our work, this was not possible due to not having all the variables to calculate those elements.

We considered in all models age and sex, given prior evidence that temporal muscle thickness varies significantly across these variables, even in healthy individuals [15,16]. This adjustment aimed to control for potential confounding and ensure that associations with nutritional biomarkers were not biased by demographic factors.

### 2.6 Statistical analysis

An initial exploratory data analysis was conducted using histograms, boxplots and others, to verify distribution, outliers, values plausibility for the variables included in the study. Descriptive analysis was performed by using central tendency measures like means and median and dispersion measures like standard deviations, interquartile range, and range for all continuous variables and percentages for categorical ones. Subsequently, a group comparison analysis was performed to assess differences between genders regarding the variables included in the study at baseline. Student´s *t*-test was used to assess these differences for continuous variables and Pearson's Chi-squared Test for categorical. For cross-sectional analyses at baseline, linear regression models were adjusted (by sex, age, height, inclusion criteria and treatment arms) to test the association between the abovementioned outcomes and TMT (exposure variable). Longitudinally, linear mixed-effects models with a random intercept for subject were performed to know the association between baseline measures for TMT and the longitudinal outcomes fitting adjusted (by sex, age, height, inclusion criteria, treatment and time) linear mixed models. For weight outcome, due to the significant associations with TMT since baseline, longitudinally, we excluded the observations at baseline and evaluated the association between TMT and weight at twelve- and twenty-four-week follow-up. Model diagnostics included visual inspection of residuals and Shapiro–Wilk tests for residual normality, and Breusch–Pagan tests when needed. Robustness was further evaluated by calculating Cook’s D to identify influential observations and by computing variance inflation factors to rule out problematic collinearity. Where a strict assumption failed, we confirmed the stability of our estimates via parametric bootstrap confidence intervals for key coefficients. We fixed the significant probability at 0.05 to evaluate the hypothesis test. The analysis was made using R Studio version 4.3.1.

## 3 Results

263 persons were screened and 206 were eligible. From those, 165 (female *n* = 84, male *n* = 81) had complete data and available images to measure TMT (Fig. 1).

At baseline, women exhibited a lower TMT (7.30 ± 1.57 vs 9.09 ± 1.65, *P* < 0.001). Descriptive and demographic information including height, weight, sex, as well as other sample characteristics are described in Table 1.

**Table 1:** Description of the study sample at baseline, overall and stratified by sex.

Variables	Overall	Female	Male	P-value
	(*n* = 165)	( *n* = 84)	(*n* = 81)	
**Age (years)**				
Mean (SD)	68.8 (5.26)	68.8 (5.75)	69.0 (4.72)	0.807
Median [Q1, Q3]	68.0 [65.0, 73.0]	67.5 [64.0, 74.0]	68.0 [66.0, 73.0]	
[MIN,MAX]	[60.0,79.0]	[60.0,79.0]	[60.0,78.0]	
**Education (years)**				
Mean (SD)	14.1 (3.21)	14.2 (3.11)	14.0 (3.32)	0.618
Median [Q1, Q3]	14.0 [12.0, 17.0]	15.0 [12.0, 17.0]	14.0 [12.0, 16.0]	
[MIN,MAX]	[7.00,22.0]	[8.00,20.0]	[7.00,22.0]	
**Inclusion criteria**				
Cardiovascular risk factors	109 (66.1 %)	57 (67.9 %)	52 (64.2 %)	0.740
Mild Cognitive impairement	56 (33.9 %)	27 (32.1 %)	29 (35.8 %)	
**Left side Temporal Muscle Thickness (TMT) (mm)**				
Mean (SD)	8.19 (1.83)	7.31 (1.54)	9.10 (1.65)	**< 0.001**
Median [Q1, Q3]	8.27 [7.21, 9.09]	7.66 [6.40, 8.47]	8.84 [8.19, 10.5]	
[MIN,MAX]	[3.82,14.2]	[3.82,10.4]	[5.03,14.2]	
**Right side Temporal Muscle Thickness (TMT) (mm)**				
Mean (SD)	8.17 (1.89)	7.30 (1.62)	9.07 (1.72)	**< 0.001**
Median [Q1, Q3]	8.26 [7.17, 9.10]	7.60 [6.45, 8.42]	8.81 [8.06, 10.1]	
[MIN,MAX]	[3.56,15.6]	[3.56,11.4]	[4.95,15.6]	
**Temporal Muscle Thickness (TMT) (mm)**				
Mean (SD)	8.18 (1.84)	7.30 (1.57)	9.09 (1.65)	**< 0.001**
Median [Q1, Q3]	8.26 [7.17, 9.09]	7.72 [6.36, 8.43]	8.83 [8.17, 10.3]	
[MIN,MAX]	[3.69,13.4]	[3.69,10.9]	[4.99,13.4]	
**Height (Cm)**				
Mean (SD)	171 (8.33)	166 (5.96)	177 (6.03)	**< 0.001**
Median [Q1, Q3]	172 [165, 178]	166 [162, 169]	178 [174, 180]	
[MIN,MAX]	[153,193]	[153,179]	[164,193]	
**Weight (Kg)**				
Mean (SD)	81.2 (14.0)	74.9 (13.5)	87.7 (11.2)	**< 0.001**
Median [Q1, Q3]	80.0 [70.0, 90.2]	74.4 [65.0, 81.6]	87.9 [79.0, 96.4]	
[MIN,MAX]	[54.2116]	[54.2116]	[62.0111]	
**Episodic Memory Quality (QEM)**				
Mean (SD)	74.0 (9.17)	74.3 (9.73)	73.6 (8.59)	0.625
Median [Q1, Q3]	75.0 [67.5, 81.7]	75.2 [67.5, 82.2]	74.2 [67.5, 79.6]	
[MIN,MAX]	[47.1,90.8]	[47.1,90.8]	[50.0,90.8]	
**Albumin (g/L)**				
Mean (SD)	39.3 (2.36)	39.3 (2.71)	39.4 (1.96)	0.840
Median [Q1, Q3]	39.3 [37.7, 41.2]	39.4 [37.6, 41.5]	39.2 [37.8, 40.8]	
[MIN,MAX]	[32.3,44.4]	[32.3,44.4]	[35.9,43.7]	
Missing	80 (48.5 %)	41 (48.8 %)	39 (48.1 %)	
**C-Reactive Protein (CRP) (mg/L)**				
Mean (SD)	2.06 (3.06)	2.57 (3.72)	1.54 (2.10)	0.118
Median [Q1, Q3]	1.40 [0, 2.50]	1.60 [0, 3.25]	1.20 [0, 2.18]	
[MIN,MAX]	[0,21.0]	[0,21.0]	[0,10.0]	
Missing	80 (48.5 %)	41 (48.8 %)	39 (48.1 %)	

**Fig. 3:**
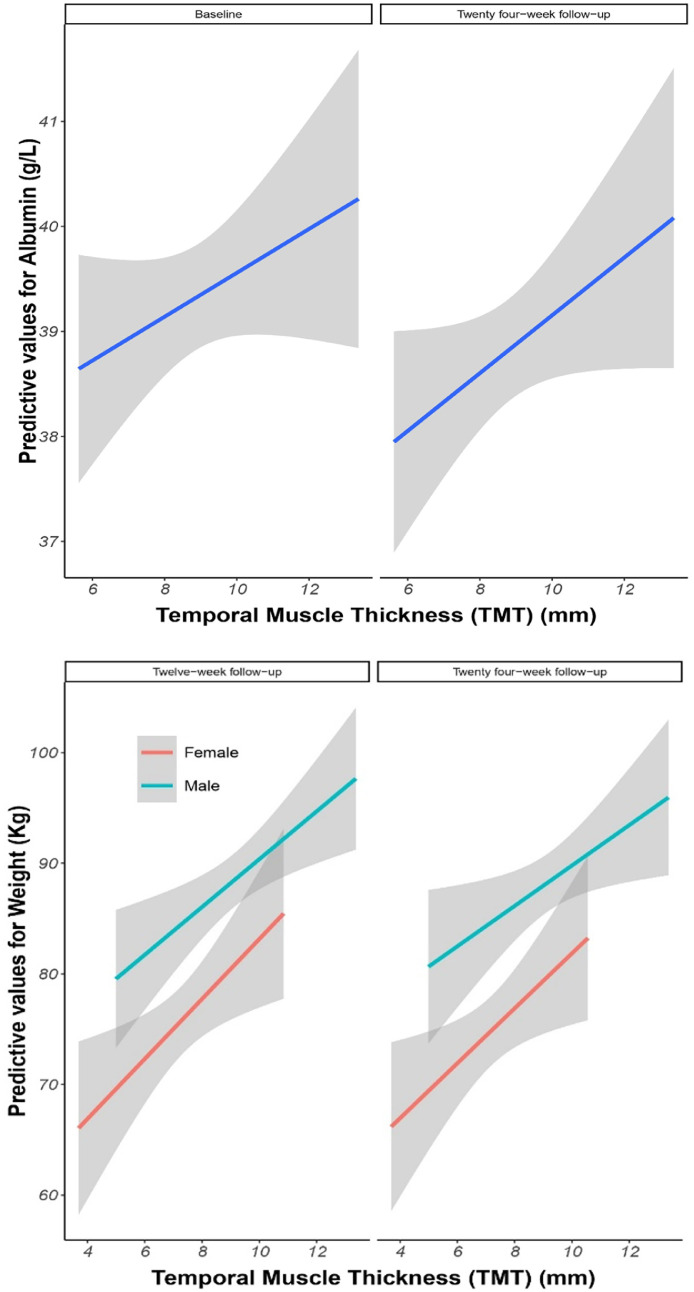
a) Association between TMT and albumin, at baseline and twenty-four-week follow-up. b) Association between TMT and weight, at twelve-week follow-up and twenty-four-week follow-up. *Albumin was not divided for sex due to smaller number participants. ** For weight outcome, due to the significant associations with TMT since baseline, we exclude the observations at baseline and evaluated the association between TMT and weight at twelve- and twenty-four-week follow-up.

At baseline, there was a positive association between weight and TMT (Estimate=1.5157, *p* = 0.009) (Table 2). In the analysis between baseline-TMT and the longitudinal outcomes at the end of the twenty-four-week follow-up, positive associations were observed between TMT and albumin levels (Estimate=0.3031, *P* = 0.048) (Fig. 3a), as well as TMT and weight (Estimate=1.8954, *P* = 0.001) (Table 2). Fig. 3b shows the association between TMT and weight, both at twelve and twenty-four-weeks, separately for men and women.

**Table 2:** Baseline and longitudinal regression between TMT and dependent variables.

Dependent variables	Estimate	Std. Error	P-value
**Baseline**			
Episodic Memory Quality (QEM)	0.1137	0.4139	0.784
Albumin (g/L)	0.2190	0.1674	0.195
Weight (Kg)	1.5157	0.5726	**0.009**
C-Reactive Protein (CRP)	−0.0200	0.2296	0.931
Creatinine	0.6934	1.1961	0.564

Longitudinals	Estimate	Std. Error	P-value
Episodic Memory Quality (QEM)	−0.1751	0.3434	0.611
Albumin (g/L)	0.3031	0.1513	**0.048**
Weight (Kg)	1.8954	0.5753	**0.001**
C-Reactive Protein (CRP)	−0.2307	0.2662	0.389
Creatinine	0.5869	1.0985	0.595

*A Analyses adjusted by sex, age, height, inclusion criteria, treatment and time.

**Baseline weight was not included in the longitudinal analysis.

There were not significant associations at baseline or longitudinally with cognition measured by the Episodic Memory Quality instrument.

## 4 Discussion

In this study, we aimed to test TMT as a marker for some of the most common nutritional status biomarkers in a population at risk of dementia. The lower the thickness of the temporal muscle at the baseline, the lower was the weight and albumin levels at 24-months. This study holds relevance due to the growing need of easily accessible biomarkers and tools that can predict outcomes in patients at risk of dementia, aiming for more effective non-pharmacological interventions.

Previously published research [9] has shown that TMT is well correlated with overall muscle mass, as well as a tool for sarcopenia detection in healthy older adults. Moreover, this idea parts from the fact that the temporal muscle, as any other muscle, undergoes age-related changes [9]. Also, Cho et al. investigated patients with probable AD, and in this population, TMT was significantly correlated with appendicular muscle mass [17]. This finding helps to translate the already mentioned findings in healthy older adults [9] to persons in the spectrum of cognitive decline.

It is important to note that TMT has been explored in the context of disease mainly as a potential biomarker with prognostic value. It was proposed as a prognostic marker in patients who suffered from subarachnoid hemorrhage (SAH), intracerebral hemorrhage or dysphagia after stroke, finding that a greater TMT is related to favorable outcomes in patients with SAH [18]. Studies in primary glioblastoma show that a greater TMT is associated with a better overall survival and progression free survival, which implicates its relevance when discussing treatment options for these individuals [19]. On the same line, an article from 2022 suggests TMT as a surrogate marker for survival and functionality in patients with amyotrophic lateral sclerosis [20].

Furthermore, TMT has also been associated with age-related important outcomes, such as cognition, gait speed, and appendicular lean soft tissue [9]. Specifically, it was found that a better performance in the Mini Mental State Examination was associated with a wider TMT [9], which is backed by extensive research that has shown a close relation between sarcopenia and cognitive decline [21]. However, in the mentioned study [9], only healthy older adults were included, and different cognitive assessment tools were employed, which could explain the discrepancy with our results.

The relationship between sarcopenia with the nutritional status is already well established [22]. Therefore, it is reasonable to consider TMT as a probable marker of nutritional status [9]. Previous research has studied the role of other muscles, such as the tongue and the masseter, as predictors of malnutrition [23]. Nevertheless, the assessment of tongue and masseter muscle volume relied on manual segmentation, which limits its practicality and clinical applicability in routine settings due to the complexity and time required. In contrast, TMT can be obtained rapidly and efficiently, requiring minimal specialized training, thereby enhancing its utility in clinical practice. In addition, TMT has also been measured with ultrasonography, in fact it has been reported that it could serve as a marker of nutritional status [24]. However, in this case, an extra exam and specialized extra knowledge to perform the ultrasonography is needed.

From a mechanistic perspective, several biological pathways could support the observed association between reduced TMT and poorer nutritional outcomes. Aging-related sarcopenia is driven in part by chronic low grade systemic inflammation that has been named "inflamm-aging" [25], and that is characterized by elevated pro-inflammatory cytokines such as TNF-α and IL‑6, which contribute to muscle catabolism and mitochondrial dysfunction. These same inflammatory processes are implicated in neurodegenerative disorders, linking peripheral muscle loss with central neuroinflammation, synaptic loss, and oxidative stress in the brain [26,27]. Moreover, the muscle–brain axis suggests that skeletal muscle secretes myokines such as irisin, which promote synaptic plasticity, neurogenesis, and cognitive function [28,29]. Malnutrition and sarcopenia together may impair neurological trophism and reduce cognitive reserve, offering a biologically plausible mechanism whereby TMT reflects both metabolic vulnerability and neuronal decline.

This framework suggests a conceptual model in which reduced TMT may signal an underlying cascade of systemic and neural decline, thus serving as an early marker of aging-related vulnerability. Future research could test whether TMT predicts the rate of progression to dementia, interacts with specific pathophysiological pathways (inflammation, neurotrophic signaling, etc.), or varies in impact depending on other variables such as subtypes of neurocognitive disorders, sex, etc.

Several limitations of the present study should be acknowledged. First, as this is a secondary analysis, the data were not originally collected with the specific aims of this manuscript in mind. The small sample size and short follow-up period limit the statistical power of our findings, which was due to different sample sizes and considering variables that were not the primary composite of the original clinical trial. Although a formal nutritional diagnosis would have been beneficial (e.g. GLIM approach), we were unable to include it due to insufficient data and the original study’s different objectives. Additionally, the sample was drawn from Norway, which may limit the generalizability of our results to other populations. As the original study was an intervention trial, we made adjustments to mitigate potential biases introduced by the intervention, as the use of purified anthocyanins was adjusted as cofounder and not considered as a study variable. Moreover, imaging was conducted at two centers with varying protocols, which may have introduced variability. We also did not investigate factors that could influence muscle volume, such as missing teeth, bruxism, or hypertonia due to missing data. Lastly, we did not include interaction terms in our models due to the relatively small sample size. Including exploratory interactions would have increased model complexity and the number of parameters to estimate, raising the risk of reduced power, convergence issues, and overfitting. We acknowledge this as a limitation and suggest that future studies with larger sample sizes explore specific interaction effects, such as age × TMT or sex × TMT.

Nonetheless, this study has several important strengths, including its longitudinal design, detailed methodology, standardized protocols, and rigorous patient selection criteria. We assess a potential novel and easily implementable methodology that could be integrated into clinical practice, hoping to improve patient management and treatment outcomes. In this sense, we believe these findings may be useful for clinicians in their practice, considering new ways of establishing dementia prognosis, and for clinical epidemiologists, highlighting outcomes in clinical trials beyond cognition.

## Conclusions

In this study, low TMT was associated with weight loss and low albumin levels after 24 weeks follow-up of patients at risk of dementia. These results highlight TMT as an accessible tool in clinical practice for outcomes beyond cognition, especially considering that most patients with cognitive decline undergo neuroimaging. TMT profiles as a potential new important variable in the clinical assessment of patients at risk of dementia, providing early insights into a poorer prognosis in nutrition.

## Data availability statement

The anonymized data collected can be requested to the main author upon request from an investigator.

## Funding information

The study funding was provided by a grant from the Norwegian Health Association (Grant no. 7330). The manufacturer of Medox®, MedPalett AS—An Evonik INDUSTRIES AG Company, is contributing by producing Medox® and placebo capsules and making them available for the study for free. Evonik, the owner of MedPalett AS—An Evonik Industries AG Company, was supporting the trial by supplying the tools for collection of feces and measurement of vascular functions and will perform the microbiome analyses. Neither Medpalett nor Evonik have had any influence on the design or conduct of the study, analysis of data, or on the decision to publish the findings or not.

Also, funded by the National Institute for Health Research (NIHR) Biomedical Research Centre at South London and Maudsley NHS Foundation Trust and King’s College London. This work was supported by NordForsk through the funding to Nordic-Japan multidomain interventions for healthy aging and prevention of dementia and disability, project number 119,886.

## Consent statement

The trial was approved by the Norwegian Regional Ethics Committee (2017/374) and registered on ClinicalTrials.gov (identifier NCT03419039). Written informed consent was obtained from all participants in accordance with Good Clinical Practice guidelines prior to their inclusion in the study.

## Declaration of Generative AI and AI-assisted technologies in the writing process

No generative AI or AI-assisted technologies were used for the preparation of this manuscript.

## CRediT authorship contribution statement

**Salomón Salazar-Londoño:** Writing – review & editing, Writing – original draft, Visualization. **Valeria Pérez-Foucrier:** Writing – review & editing, Writing – original draft, Visualization. **Jonathan Patricio Baldera:** Writing – review & editing, Formal analysis. **Markus Aarsland:** Writing – review & editing, Visualization, Methodology. **Luis Carlos Venegas-Sanabria:** Writing – review & editing, Visualization, Methodology. **Miguel German Borda:** Writing – review & editing, Visualization, Methodology, Formal analysis, Conceptualization.

## Declaration of competing interest

The authors declare that they have no known competing financial interests or personal relationships that could have appeared to influence the work reported in this paper.

## Appendix Supplementary materials

Image, application 1
