# Supplementary material for: Temporal muscle thickness predicts change in nutritional markers in individuals at risk of dementia: Insights from a 24-week longitudinal study
Source: JAR Life. 2025 Aug 8;14:100023. doi: 10.1016/j.jarlif.2025.100023 (PMC12357314; doi:10.1016/j.jarlif.2025.100023)
Supplement: Supplementary file 1 [file mmc1.docx]

**Table 1. Comparison between missing cases and observed cases (included in the final sample for this analysis).**

|  | **missing cases** | **Observed cases** | **Overall** | **P-value** |
| --- | --- | --- | --- | --- |
|  | **(n = 36)** | **(n = 165)** | **(n = 201)** |  |
| **Gender** |  |  |  |  |
| Female | 17 (47.2%) | 84 (50.9%) | 101 (50.2%) | 0.689 |
| Male | 19 (52.8%) | 81 (49.1%) | 100 (49.8%) |  |
| **Age** |  |  |  |  |
| Mean (SD) | 69.2 (5.01) | 68.8 (5.26) | 68.9 (5.20) | 0.712 |
| Median [Q1, Q3] | 69.0 [65.8, 74.0] | 68.0 [65.0, 73.0] | 68.0 [65.0, 73.0] |  |
| [MIN,MAX] | [60.0,78.0] | [60.0,79.0] | [60.0,79.0] |  |
| **Education (years)** |  |  |  |  |
| Mean (SD) | 13.9 (3.21) | 14.1 (3.21) | 14.1 (3.20) | 0.684 |
| Median [Q1, Q3] | 14.0 [11.0, 17.0] | 14.0 [12.0, 17.0] | 14.0 [12.0, 17.0] |  |
| [MIN,MAX] | [9.00,22.0] | [7.00,22.0] | [7.00,22.0] |  |
| **Treatment** |  |  |  |  |
| Intervention | 17 (47.2%) | 86 (52.1%) | 103 (51.2%) | 0.594 |
| Control | 19 (52.8%) | 79 (47.9%) | 98 (48.8%) |  |
| **Inclusion criteria** |  |  |  |  |
| Cardiovascular risk factors | 27 (75.0%) | 109 (66.1%) | 136 (67.7%) | 0.299 |
| Mild Cognitive impairment | 9 (25.0%) | 56 (33.9%) | 65 (32.3%) |  |
| **Height (Cm)** |  |  |  |  |
| Mean (SD) | 172 (9.48) | 171 (8.33) | 171 (8.53) | 0.767 |
| Median [Q1, Q3] | 171 [165, 179] | 172 [165, 178] | 172 [165, 178] |  |
| [MIN,MAX] | [153,185] | [153,193] | [153,193] |  |
| **Weight (Kg)** |  |  |  |  |
| Mean (SD) | 82.5 (15.0) | 81.2 (14.0) | 81.4 (14.1) | 0.637 |
| Median [Q1, Q3] | 83.6 [69.8, 90.9] | 80.0 [70.0, 90.2] | 80.0 [70.0, 90.5] |  |
| [MIN,MAX] | [52.5,110] | [54.2,116] | [52.5,116] |  |
| **Episodic Memory Quality (QEM)** |  |  |  |  |
| Mean (SD) | 73.5 (9.35) | 74.0 (9.17) | 73.9 (9.18) | 0.804 |
| Median [Q1, Q3] | 75.0 [69.2, 80.1] | 75.0 [67.5, 81.7] | 75.0 [67.5, 80.4] |  |
| [MIN,MAX] | [51.3,88.3] | [47.1,90.8] | [47.1,90.8] |  |
| **Albumin *** |  |  |  |  |
| Mean (SD) | 38.4 (4.25) | 39.3 (2.36) | 39.2 (2.63) | 0.512 |
| Median [Q1, Q3] | 39.1 [37.6, 40.8] | 39.3 [37.7, 41.2] | 39.3 [37.7, 41.2] |  |
| [MIN,MAX] | [27.1,43.0] | [32.3,44.4] | [27.1,44.4] |  |
| **C-Reactive Protein (CRP) *** |  |  |  |  |
| Mean (SD) | 1.84 (1.85) | 2.06 (3.06) | 2.04 (2.93) | 0.728 |
| Median [Q1, Q3] | 1.65 [0, 2.63] | 1.40 [0, 2.50] | 1.40 [0, 2.50] |  |
| [MIN,MAX] | [0,6.20] | [0,21.0] | [0,21.0] |  |
| **Creatinine *** |  |  |  |  |
| Mean (SD) | 79.1 (13.2) | 77.8 (17.4) | 78.0 (16.9) | 0.767 |
| Median [Q1, Q3] | 80.5 [73.8, 88.3] | 76.0 [66.0, 87.0] | 77.0 [67.0, 87.0] |  |
| [MIN,MAX] | [51.0,98.0] | [50.0,147] | [50.0,147] |  |
| *** Sub-group analysis** |  |  |  |  |
